# Traditional Chinese medicine formulation Yanggan Jiedu Sanjie inhibits TGF-β1-induced epithelial-mesenchymal transition and metastatic potential in human hepatocarcinoma Bel-7402 cells

**DOI:** 10.1186/s12906-019-2477-9

**Published:** 2019-03-15

**Authors:** Bing Hu, Hong-Mei An, Xia Yan, Jia-Lu Zheng, Xiao-Wei Huang, Miao Li

**Affiliations:** 1 Institute of Traditional Chinese Medicine in Oncology, Department of Oncology, Longhua Hospital, Shanghai University of Traditional Chinese Medicine, Shanghai, 200032 People’s Republic of China; 2 Department of Science & Technology, Longhua Hospital, Shanghai University of Traditional Chinese Medicine, Shanghai, 200032 People’s Republic of China

**Keywords:** Hepatocellular carcinoma, Epithelial-mesenchymal transition, Chinese herbal medicine, Metastasis

## Abstract

**Background:**

Epithelial-mesenchymal transition (EMT) is a vital process in cancer progression and metastasis. Yanggan Jiedu Sanjie (YGJDSJ) is Traditional Chinese Medicine formulation for liver cancer treatment. In the present study, we evaluated the effects of YGJDSJ on TGF-β1-induced EMT in hepatocellular carcinoma Bel-7402 cells.

**Methods:**

Bel-7402 cells were treated with TGF-β1 and YGJDSJ. EMT was identified by morphological changes and expression of marker proteins. Cell morphology was observed under a microscope. Protein expression and phosphorylation was detected by western blotting. Cell migration was measured by the scratch assay. Cell adhesion and invasion was detected by a commercial kit.

**Results:**

YGJDSJ reversed TGF-β1-induced morphological changes, as well as the expression of the EMT markers E-cadherin and N-cadherin in Bel-7402 cells. YGJDSJ also inhibited TGF-β1 up-regulated Smad3 phosphorylation and Snail expression in Bel-7402 cells. Moreover, YGJDSJ inhibited TGF-β1-induced cell adhesion, migration and invasion in Bel-7402 cells.

**Conclusions:**

YGJDSJ inhibited TGF-β1-induced EMT and mediated metastatic potential of Bel-7402 cells, which may be related to down-regulation of Smad3 phosphorylation and Snail expression. The present study provides a new basis for application of this herbal formula for prevention of liver cancer metastasis.

## Background

Liver cancer ranks sixth in incidence and fourth in mortality among all cancers globally [1]. Hepatocellular carcinoma (HCC) is the most common liver cancer (75–85%) [1]. Surgery is the only curative treatment option for early HCC [2, 3]. HCC is prone to metastasize to lung (39.5–53.8%), lymph node (33.8–34.2%), bone (25.4–38.5%) and other sites through the lymph or blood circulation [4, 5]. The efficacy of current treatment for metastatic HCC, including radiotherapy, chemotherapy and targeted therapy, is very poor [6, 7]. Therefore, prevention of HCC metastasis is particularly important.

HCC metastasis is closely related to epithelial-mesenchymal transition (EMT) [8, 9]. EMT is a biological process in which epithelial cells lose their epithelial characteristics and acquire the phenotype of mesenchymal cells, including changes in cell morphology and expression of marker proteins, such as down-regulation of epithelial marker gene E-cadherin and up-regulation of mesenchymal marker gene N-cadherin [8, 9]. Tumor cells with EMT have increased motility and invasiveness, which facilitate their metastasis. Suppression of EMT could inhibit HCC metastasis [10, 11].

Yanggan Jiedu Sanjie (YGJDSJ) is a modern Chinese herbal formula developed by us for the treatment of HCC [12]. YGJDSJ composed of multiple herbs. Some herbs in YGJDSJ have demonstrated inhibitory effects on EMT in cancer cells. *Solanum nigrum* L. (Long-Kui) and its ingredient α-Solanine, polysaccharide of *Scutellaria barbata* D. Don (Ban-Zhi-Lian), *Duchesnea indica* (Andr.) Focke (She-Mei), and β-elemene (a compound of *Curcuma wenyujin* Y.H. Chen et C. Ling (Yü-Jin)) can inhibit EMT in various cancer cells [13–17]. These findings suggested that YGJDSJ may also have a similar effect on EMT. In this study, we evaluated the effect of YGJDSJ on transforming growth factor-β1 (TGF-β1)-induced EMT in human HCC Bel-7402 cells.

## Materials and methods

### Chemicals and reagents

DMEM medium and fetal bovine serum were purchased from Thermo Fisher Scientific (Waltham, MA). TGF-β1 was obtained from PeproTech (Rocky Hill, NJ). Antibodies against Smad3, p-Smad3 (Ser423/425), Snail and GAPDH were from Cell Signaling Technology (Danvers, MA). E-cadherin and N-cadherin antibodies were bought from Santa Cruz Biotechnology (Santa Cruz, CA). CytoSelect™ 48-Well Cell Adhesion Assay and CytoSelect™ 24-Well Cell Invasion Assay kits were produced by Cell Biolabs (San Diego, CA).

#### YGJDSJ extraction

The herbs in YGJDSJ formula (Chinese patent No. ZL201110145109.0) are the fruits of *Ligustrum lucidum* Ait. (Nü-zhen-zi) 12 g, *D. indica* (Andr.) Focke (She-Mei) 15 g, *S. nigrum* L. (Long-Kui) 15 g, *S. barbata* D. Don (Ban-Zhi-Lian) 30 g, *Euphorbia helioscopia L.* (Ze-Qi) 15 g, the root of *Ranunculus ternatus* Thunb. (Mao-Zhua-Cao) 15 g, the root of *C. wenyujin* Y. H. Chen et C. Ling (Yü-Jin) 15 g and the root of *Polygonum cuspidatum* Sieb. et Zucc. (Hu-Zhang) 15 g. All herbs were obtained from the dispensary of Chinese medicine of Longhua Hospital and identified by Professor Liwen Xu from Shanghai University of Traditional Chinese Medicine, Shanghai, China. Voucher specimen is deposited in Institute of Traditional Chinese Medicine in Oncology, Longhua Hospital, Shanghai University of Traditional Chinese Medicine, Shanghai, China (specimen number: TCM-HCC-001). YGJDSJ extraction has been described previously [12]. YGJDSJ extract were dissolved in PBS and stored at − 20 °C until further use.

### Cell culture

Bel-7402 cells were obtained from The Cell Bank of Type Culture Collection of Chinese Academy of Sciences (CBTCCCAS) and checked by CBTCCCAS. The cells were cultured in DMEM medium containing 10% FBS and 1% Pen-Strep, and maintained at 37 °C in a humidified atmosphere with 5% CO_2_.

### EMT induction

Bel-7402 cells (5 × 10^5^) in logarithmic growth phase were inoculated in 6-well plates and cultured in serum free DMEM and allowed to attach for 24 h before treatment. The cells were then treated with TGF-β1 (10 ng/mL) and YGJDSJ (100 μg/mL) or same volume of PBS for 48 h. The morphology of the cells was observed under a microscope.

### Scratch / migration assay

Cell migration was measured by the scratch assay [18, 19]. Bel-7402 cells (1 × 10^6^) were incubated in 6-well plates and cultured to 95% confluency. Then the cells were scratched by a sterile pipette tip, and washed three times with PBS. Fresh medium was added and the cells were treated with TGF-β1 (10 ng/mL) and YGJDSJ (100 μg/mL) or equal volume of PBS for 48 h. The cell migration was observed by microscopy.

### Cell adhesion assay

Cell adhesion was detected by a commercial kit according to the manufacturer’s manual. Briefly, 1 × 10^5^ TGF-β1 and YGJDSJ treated or untreated Bel-7402 cells were added to a 48 well plate. TGF-β1 (10 ng/mL), YGJDSJ (100 μg/mL) or same volume of PBS was also added to the wells. Cells were incubated for 90 min at 37 °C and stained with staining solution for 10 min at room temperature. After aspirating the staining solution, the plate was gently washed three times with 500 μl deionized water and air dried. 200 μL of extraction solution was then added to the wells and incubated for 10 min. The optical density of each well was measured at OD 560 nm by a plate reader.

### Cell invasion assay

Cell invasion was detected by a commercial kit according to the manufacturer’s protocol. Briefly, 3 × 10^5^ TGF-β1 and YGJDSJ treated or untreated Bel-7402 cells were added to the inner side of cell insert, and 500 μL of DMEM media with 10% FBS was added to the lower well of the invasion plate. TGF-β1 (10 ng/mL), YGJDSJ (100 μg/mL) or same volume of PBS was also added to the wells. The cells were incubated for 12 h at 37 °C. After removing the non-invasive cells, the inserts were stained with staining solution for 10 min at room temperature, and observed under a microscope. 200 μL of extraction solution was then added to the wells and incubated for 10 min. The optical density of each well was measured at OD 560 nm by a plate reader.

### Western blot

Western blotting was performed as previously described [20, 21]. Briefly, the cells were lysed and subjected to 8–12% SDS-PAGE electrophoresis, and then transferred onto a nitrocellulose membrane (Bio-Rad, Richmond, CA). The membrane was blocked with 5% non-fat milk, washed, and probed with antibodies against E-cadherin (1:200), N-cadherin (1:200), Smad3 (1:1000), p-Smad3 (1:1000), Snail (1:1000) or GAPDH (1:2000) at 4 °C overnight. The blots were then washed and incubated with secondary antibodies (1:4000), developed by ECL substrates and exposed by ChemiDoc™ Touch Imaging System (Bio-Rad, Hercules, CA). Proteins expression was quantified by the Quantity One software (Bio-Rad, Hercules, CA).

### Statistical analyses

Results are expressed as means ± standard deviation of at least two independent experiments. Differences between different treatment groups were analyzed by one-way ANOVA. Differences were considered significant at *p*-values < 0.05.

## Results

### YGJDSJ reversed TGF-β1-induced morphological changes

**Fig. 1:**
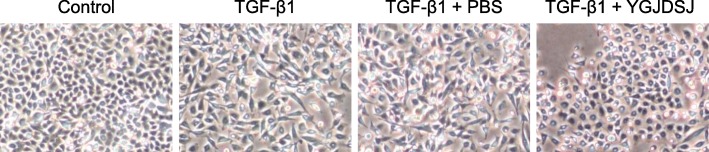
Effects of YGJDSJ on TGF-β1-induced morphological changes. Bel-7402 cells were treated with TGF-β1 and YGJDSJ or PBS for 48 h, and cell morphology was observed under a microscope (× 200)

After TGF-β1 treatment, the morphology of Bel-7402 cells changed from round or oval to spindle shaped. The distribution of the cells was looser and they exhibited mesenchymal morphology. After YGJDSJ treatment, Bel-7402 cells were reverted to epithelial morphology, which was round or oval (Fig. 1).

### YGJDSJ reversed TGF-β1-induced expression change of EMT marker proteins

**Fig. 2:**
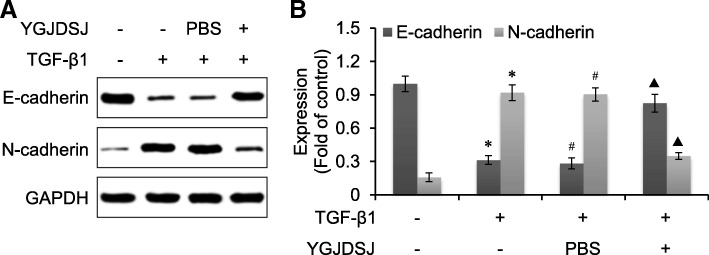
Effects of YGJDSJ on TGF-β1-induced expression change of EMT marker proteins. **a**, Bel-7402 cells were treated with TGF-β1 and YGJDSJ or PBS for 48 h, and subjected to western blotting using indicated antibodies. **b**, protein expression was quantified by the Quantity One software. ^*^p < 0.01, versus control group; ^#^*p* > 0.05, versus TGF-β1 group; ^▲^p < 0.01, versus PBS group

Western blot analysis showed that the expression of epithelial marker gene E-cadherin was down-regulated, while the mesenchymal marker gene N-cadherin was up-regulated after TGF-β1 treatment. YGJDSJ increased E-cadherin expression and inhibited N-cadherin expression (*p* < 0.01) (Fig. 2). These observations suggested that YGJDSJ inhibited TGF-β1-induced EMT in Bel-7402 cells.

### YGJDSJ inhibited TGF-β1-induced Smad3 phosphorylation

**Fig. 3:**
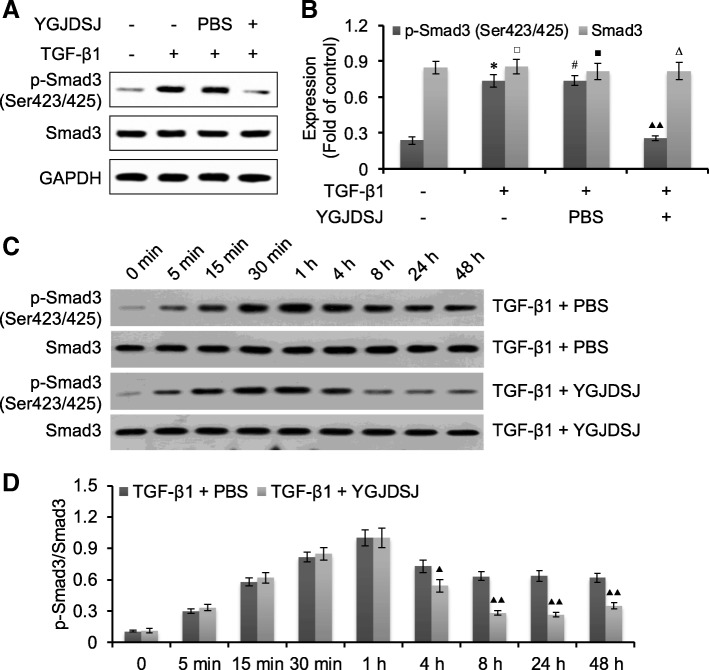
Effects of YGJDSJ on TGF-β1-induced Smad3 phosphorylation. **a**, Bel-7402 cells were treated with TGF-β1 and YGJDSJ or PBS for 48 h, and subjected to western blotting using indicated antibodies. **c**, Bel-7402 cells were treated with TGF-β1 and YGJDSJ or PBS for indicated times, and subjected to western blotting using Samd3 and p-Smad3. **b** and **d**, protein expression was quantified by the Quantity One software. ^*^p < 0.01, versus control group; ^#^*p* > 0.05, versus TGF-β1 group; ^▲^*p* < 0.05, versus PBS group; ^▲▲^*p* < 0.01, versus PBS group; ^□^*p* > 0.05, versus control group; ^■^*p* > 0.05, versus TGF-β1 group; ^∆^*p* > 0.05, versus PBS group

It has been reported that Smad3 is involved in TGF-β1 induced EMT [22, 23]. We observed Smad3 expression and phosphorylation by western blot. The results showed that the expression of Smad3 did not change significantly; but the phosphorylation levels of Smad3 were increased after TGF-β1 treatment. YGJDSJ could inhibit TGF-β1-induced phosphorylation of Smad3 (*p* < 0.01) (Fig. 3).

### YGJDSJ inhibited TGF-β1-induced Snail expression

**Fig. 4:**
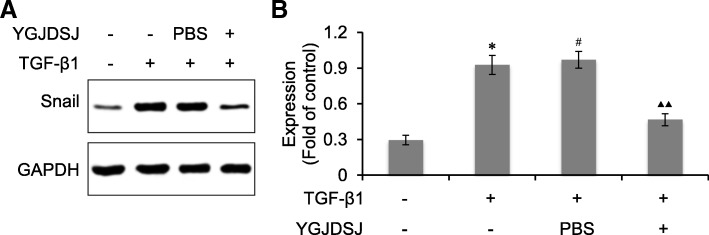
Effects of YGJDSJ on TGF-β1-induced Snail expression. **a**, Bel-7402 cells were treated with TGF-β1 and YGJDSJ or PBS for 48 h, and subjected to western blotting using indicated antibodies. **b**, protein expression was quantified by the Quantity One software. ^*^p < 0.01, versus control group; ^#^*p* > 0.05, versus TGF-β1 group; ^▲▲^*p* < 0.01, versus PBS group

Snail is an important transcriptional regulator of EMT that can be up-regulated by TGF-β1 [24, 25]. We also detected the expression of Snail. The results showed that the expression of Snail in Bel-7402 cells was up-regulated by TGF-β1. YGJDSJ could inhibit the expression of Snail induced by TGF-β1 (*p* < 0.01) (Fig. 4).

### YGJDSJ inhibited TGF-β1-induced cell adhesion

**Fig. 5:**
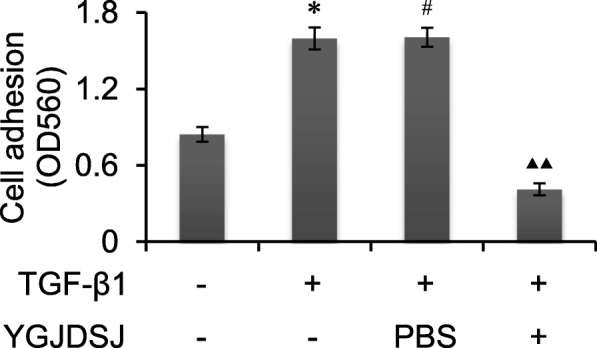
Effects of YGJDSJ on TGF-β1-induced cell adhesion. Bel-7402 cells were treated with TGF-β1 and YGJDSJ or PBS for 48 h, and subjected to cell adhesion assay using a commercial kit according to the manufacturer’s manual. ^*^p < 0.01, versus control group; ^#^*p* > 0.05, versus TGF-β1 group; ^▲▲^*p* < 0.01, versus PBS group

Cell adhesion is an important biological process in tumor metastasis, and EMT can promote tumor cell adhesion [26, 27]. The results showed that the adhesion ability of Bel-7402 cells was enhanced by TGF-β1. YGJDSJ could inhibit TGF-β1-induced cell adhesion in Bel-7402 cells (p < 0.01) (Fig. 5).

### YGJDSJ inhibited TGF-β1-induced cell migration

**Fig. 6:**
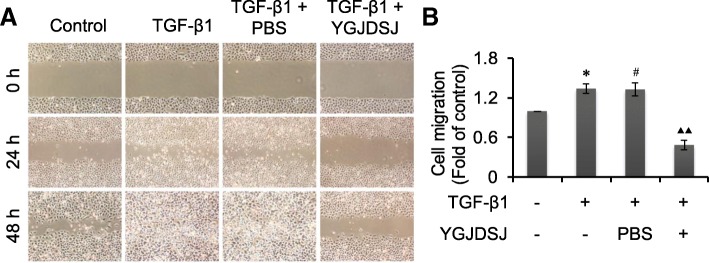
Effects of YGJDSJ on TGF-β1-induced cell migration. **a**, Bel-7402 cells were scratched by a sterile pipette tip and treated with TGF-β1 and YGJDSJ or PBS for 48 h, cell migration was observed under a microscope (× 40). **b**, Cell migration distance at 24 h were measured by electronic ruler and expressed as fold of control. ^*^p < 0.01, versus control group; ^#^*p* > 0.05, versus TGF-β1 group; ^▲▲^*p* < 0.01, versus PBS group

Scratch assay is a simple and convenient method to detect cell migration [18, 19]. Results from scratch assay showed that the migration ability of Bel-7402 cells was enhanced by TGF-β1; but YGJDSJ inhibited the cell migration induced by TGF-β1 (Fig. 6).

### YGJDSJ inhibited TGF-β1-induced cell invasion

**Fig. 7:**
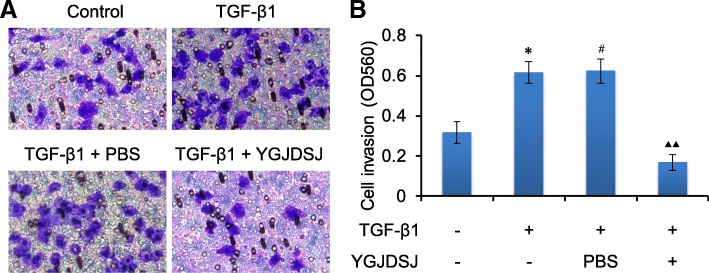
Effects of YGJDSJ on TGF-β1-induced cell invasion. Bel-7402 cells were treated with TGF-β1 and YGJDSJ or PBS for 48 h, and subjected to cell invasion assay using a commercial kit according to the manufacturer’s protocol. Cell invasion was observed under a microscope (× 200) (**a**) and measured at OD 560 nm by a plate reader (**b**). ^*^p < 0.01, versus control group; ^#^*p* > 0.05, versus TGF-β1 group; ^▲▲^*p* < 0.01, versus PBS group

Cell invasion is an important process in tumor metastasis, and EMT can promote the invasion of tumor cells [28, 29]. Transwell assay showed that the invasion ability of Bel-7402 cells was enhanced by TGF-β1. YGJDSJ could inhibit the invasion of Bel-7402 cells induced by TGF-β1 (*p* < 0.01) (Fig. 7).

## Discussion

YGJDSJ is composed of several Chinese herbs with anticancer effects. The fruits of *L. lucidum* Ait. (Nü-Zhen-Zi) inhibits proliferation, as well as promotes apoptosis and senescence in HCC cells [30]. *S. barbata* D. Don (Ban-Zhi-Lian) promotes apoptosis of HCC cells via mitochondrial pathway [31]. *S. nigrum* L. inhibits proliferation, induces apoptosis, and arrests cell cycle at G2/M phase in HCC cells [32]. *D. indica* (Andr.) Focke (She-Mei) inhibits proliferation, induces apoptosis, and arrests cell cycle in cervical cancer cells [33].

*E. helioscopia* L. (Ze-Qi) inhibits tumor growth, induces apoptosis, and inhibits metastasis in HCC [34]. *R. ternatus* Thunb. (Mao-Zhao-Cao) inhibits proliferation and tumor growth in HCC [35, 36]. *C. wenyujin* Y.H. Chen et C. Ling (Yü-Jin) has a wide range of anticancer effects, its main components include β-elemene and curcumin [37–39]. *P. cuspidatum* Sieb. et Zucc (Hu-Zhang) exhibits anticancer effect on various tumors and resveratrol is one of its important ingredients [39, 40]. Thus, YGJDSJ is a modern anticancer herbal formula.

Some herbs or compounds in YGJDSJ have showed inhibitory effects on EMT. *S. barbata* (Ban-Zhi-Lian) polysaccharide inhibits EMT in colon cancer cells [15]. *D. indica* (She-Mei) extract inhibits migration and EMT in lung adenocarcinoma cells [16]. *S. nigrum* (Long-Kui) suppresses EMT in breast cancer cells [13]. β-elemene, a compound from *C. wenyujin* Y.H. Chen et C. Ling (Yü-Jin), inhibits EMT in glioblastoma and breast cancer cells [17, 41]. Resveratrol, a component of *P. cuspidatum* Sieb. et Zucc. (Hu-Zhang), inhibits EMT in multiple cancer cells [42–44].

TGF-β1 is a cytokine that is associated with multiple bioprocess, such as cell proliferation, differentiation, death and EMT, and is frequently used for EMT induction [8, 22, 23, 27, 42]. In present study, we observed that upon TGF-β1 treatment, the morphology of Bel-7402 cells changed from oval epithelial morphology to spindle mesenchymal morphology with down-regulation of E-cadherin and up-regulation of N-cadherin, indicating the occurrence of EMT. On the other hand, YGJDSJ reversed TGF-β1-induced morphological changes, as well as the expression of the EMT markers E-cadherin and N-cadherin in Bel-7402 cells, suggesting that YGJDSJ could inhibit EMT induced by TGF-β1.

TGF-β1 binding to its receptor could recruit and phosphorylate Smads, and up-regulate Snail, thus leading to EMT [8, 45–47]. Snail is an EMT-related transcriptional repressor, which regulate the transcription of target genes, such as E-cadherin, and is related to cancer metastasis [24, 25]. Snail is required for TGF-β1-induced EMT [48, 49]. Natural products, such as Jianpi Huayu Decoction, Nobiletin and Resveratrol, could inhibit EMT by inhibiting TGF-β1/Smad signaling and Snail [50–52]. Our results showed that Smad3 phosphorylation and Snail expression were increased in Bel-7402 cells by TGF-β1, suggesting that EMT induced by TGF-β1 was associated with Smad3 and Snail. YGJDSJ could inhibit TGF-β1-induced EMT, and decrease Smad3 phosphorylation and Snail expression, suggesting that Smad3 and Snail were involved in the effect of YGJDSJ on EMT.

EMT causes cancer cells to acquire mesenchymal phenotype, enhance their adhesion, migration and invasion capacities, thus promoting tumor metastasis [27–29, 53]. The present study showed that TGF-β1-induced EMT could enhance cell adhesion, migration and invasion of Bel-7402 cells. YGJDSJ inhibited cell adhesion, migration and invasion induced by TGF-β1, suggesting that YGJDSJ can inhibit metastatic potential of Bel-7402 cells by inhibiting EMT.

## Conclusions

In conclusion, YGJDSJ inhibits TGF-β1-induced EMT and cell adhesion, migration and invasion in Bel-7402 cells, which is related to down-regulation of Smad3 phosphorylation and Snail expression. The present study provides a new basis for the application of YGJDSJ for the prevention and treatment of HCC metastasis, which is worthy of further study.

## Abbreviations

ANOVA: Analysis of variance
CBTCCCAS: The Cell Bank of Type Culture Collection of Chinese Academy of Sciences
DMEM: Dulbecco’s modified Eagle’s medium
ECL: Enhanced chemiluminence
EMT: Epithelial-mesenchymal transition
FBS: Fetal bovine serum
GAPDH: Glyceraldehyde-3-phosphate dehydrogenase
HCC: Hepatocellular carcinoma
PBS: Phosphate-buffered saline
SDS-PAGE: Sodium dodecyl sulfate-polyacrylamide gel electrophoresis
TGF-β1: Transforming growth factor-β1
YGJDSJ: Yanggan Jiedu Sanjie herbal formula
